# Identification and analysis of short-term and long-term salt-associated lncRNAs in the leaf of *Avicennia marina*

**DOI:** 10.1186/s12870-024-05216-z

**Published:** 2024-06-05

**Authors:** Lingling Wang, Yixuan Fu, Zixin Yuan, Jingyi Wang, Yali Guan

**Affiliations:** 1 Ministry of Education Key Laboratory for Ecology of Tropical Islands, Key Laboratory of Tropical Animal and Plant Ecology of Hainan Province, College of Life Sciences, Hainan Normal University, Haikou, 571158 China; 2 Hainan Observation and Research Station of Dongzhaigang Mangrove Wetland Ecosystem, Haikou, 571158 China

**Keywords:** LncRNA, *Avicennia marina*, Mangrove, Salt stress, Leaf specificity

## Abstract

As a highly salt-resistant mangrove, *Avicennia marina* can thrive in the hypersaline water. The leaves of *Avicennia marina* play a crucial role in salinity stress adaptability by secreting salt. Although the functions of long non-coding RNAs (lncRNAs) in leaves remain unknown, they have emerged as regulators in leaf development, aging and salt response. In this study, we employed transcriptomic data of both short-term and long-term salt treated leaves to identify salt-associated lncRNAs of leaf tissue. As a result, 687 short-term and 797 long-term salt-associated lncRNAs were identified. Notably, both short-term and long-term salt-associated lncRNAs exhibited slightly longer lengths and larger exons, but smaller introns compared with salt-non-associated lncRNAs. Furthermore, salt-associated lncRNAs also displayed higher tissue-specificity than salt-non-associated lncRNAs. Most of the salt-associated lncRNAs were common to short- and long-term salt treatments. And about one fifth of the downregulated salt-associated lncRNAs identified both in two terms were leaf tissue-specific lncRNAs. Besides, these leaf-specific lncRNAs were found to be involved in the oxidation–reduction and photosynthesis processes, as well as several metabolic processes, suggesting the noticeable functions of salt-associated lncRNAs in regulating salt responses of *Avicennia marina* leaves.

**Supplementary Information:**

The online version contains supplementary material available at 10.1186/s12870-024-05216-z.

## Background

In eukaryotes, transcriptome studies have revealed that over 90% of their genomes was transcribed. However, only approximately 2% of the transcribed genome possesses protein-coding ability, and most of the transcripts are non-coding RNAs (ncRNAs) [1, 2]. The ncRNAs are involved in a series of biological processes and pathways in organisms by acting on corresponding targets, such as DNA, RNA, and proteins [3, 4]. Long non-coding RNAs (lncRNAs) are freshly discovered functional ncRNAs that are longer than 200 nt in length, possessing little or no coding possibility, exerting extensive regulatory effects through a variety of mechanisms [5–7]. Although lncRNAs share poor sequence conservations among species compared with coding genes, emerging evidence indicates that lncRNAs play significant roles in different biological processes [8–13].

To date, with the datasets produced by the popularization of sequencing technology, as well as the rapidly developing and extensively utilizing of bioinformatics analysis, numerous of lncRNAs have been identified in plant species, including Arabidopsis [14, 15], rice [6, 13], wheat [16], soybean [17, 18], tomato [19], *Brassica* [11], maize [20], rubber [21, 22], etc.. To a certain extent, some of these plant lncRNAs have been demonstrated to exhibiting differential expression patterns in abiotic and biotic stress responses [23]. In Arabidopsis, the lncRNA *SABC1* (*salicylic acid biogenesis controller 1*) suppresses salicylic acid production and plant immunity by negatively regulating the transcriptions of *NAC3* and *ICS1* (*isochorismate synthase 1*) [24]. *DRIR* (*Drought induced lncRNA*), a lncRNA that presents relatively low expression level under normal condition but can be markedly activated by salt and drought stresses as well as ABA treatment, and then positively regulates plant abiotic stress responses by controlling the expression of couples of stress-responsive genes [25]. Additionally, a lncRNA, *cis*-*NATZmNAC48* is involved in drought stress response and may decrease *ZmNAC48*’s expression level to affect the closure of stomata in maize [26]. Besides, *Ptlinc-NAC72* is prominently induced by long-term salt treatment and overexpressing of it confers salt-hypersensitive phenotype in *Populus* [27]. Although the stress-responsive functions of lncRNAs have been clarified in several plant species, the roles of lncRNAs in regulating salt stress response in leaf tissue still remains largely unknown, especially in *Avicennia marina*.

*Avicennia marina* is a kind of mangrove that can thrive in the hypersaline environment, preserve the biodiversity and ecological environment of coastline. While the mechanisms and regulators referred to salinity stress response in *Avicennia marina* are poorly understood. In this study, we utilized RNA sequencing data of both short-term and long-term salt-treated leaves to identify and characterize salt-associated lncRNAs (SA-lncRNAs). Following, we conducted a comprehensive functional analysis of salt-associated lncRNAs in *Avicennia marina* under various salinity treatments. This research aims to provide a better understanding of the lncRNAs’ function under the salinity stress in leaf tissue, and contribute to our background knowledge about their regulatory roles. Ultimately, identify salt-responsive regulators as well as provide us new insights to understand the salt-responsive mechanisms of *Avicennia marina* as well as other plant species.

## Materials and methods

### Identification of salt-associated lncRNAs in the leaf tissue of *Avicennia marina*

We employed the RNA sequencing data from the leaves of *Avicennia marina* under diverse salinity treatments, including 0 mM NaCl (Control, CK), 500 mM NaCl for 24 h (24 h) and 48 h (48 h), as well as 250 mM NaCl for 14 days (14d) and 28 days (28d) to respectively identify the short-term (24 h and 48 h) and long-term (14d and 28d) salt-associated lncRNAs [28]. The salt-associated lncRNAs were identified by comparing 24 h, 48 h, 14d, 28d group (treatment group) with the control group (CK), separately. These transcriptomic datasets were obtained from NCBI SRA, then the software Salmon (version1.4.0) was employed to align the reads to transcripts and compute raw read counts for each transcript [29]. Subsequently, we performed data normalization using the Trimmed Mean of the M-values (TMM) approach and transformed the counts into FPKM (Fragments Per Kilobase of sequence per Million mapped reads) values using edgeR (version 3.30.3) [30].

The transcripts of lncRNAs were obtained from our previous study [31] according to the characteristics of lncRNAs, including the sequence length (> 200 bp), exon number > 1, sharing no exon overlap with mRNAs, and no protein coding potentiality as previously described [22]. Secondly, for the above obtained lncRNAs, if one lncRNA is with log2FoldChange of 24hvsCK > 1 & log2FoldChange of 48hvsCK > 0 or log2FoldChange of 24hvsCK > 0 & log2FoldChange of 48hvsCK > 1, the lncRNA is defined as the upregulated short-term SA-lncRNA (salt-associated lncRNA). While if one lncRNA is with log2FoldChange of 24hvsCK < -1 & log2FoldChange of 48hvsCK < 0 or log2FoldChange of 24hvsCK < 0 & log2FoldChange of 48hvsCK < -1, the lncRNA is defined as the downregulated short-term SA-lncRNA. Similarly, once the lncRNA is with log2FoldChange > 0 both in 14dvsCK and 28dvsCK and log2FoldChange > 1 at least in one group, this lncRNA is reckoned as the upregulated long-term salt-associated lncRNA (long-term SA-lncRNA). The lncRNA with log2FoldChange < 0 both in 14dvsCK and 28dvsCK and log2FoldChange < -1 at least in one group is recognized as the downregulated long-term SA-lncRNA. Both of the upregulated and downregulated long-term and short-term SA-lncRNAs are regarded as the salt-associated lncRNAs. Meanwhile, others are salt-non-associated lncRNAs (SNA-lncRNAs).

### Chromosome distributions of the salt-associated and salt-non-associated leaf-specific lncRNAs

To examine the distributions of SNA-lncRNAs and SA-lncRNAs across the chromosomes, we separately counted the total number of short-term and long-term SA-lncRNAs as well as SNA-lncRNAs for each chromosome. In order to view their detail chromosomal distributions, we subsequently partitioned each chromosome into 200 kb-width bins from the initial position to the end with 200 kb step and calculated the number of salt-associated and salt-non-associated lncRNAs in each bin [22].

### Characteristics of salt-associated and salt-non-associated leaf lncRNAs in *Avicennia marina*

The exon number, size, length, etc. of SA-lncRNAs and SNA-lncRNAs were calculated with R software (version 4.0.2, https://cran.r-project.org) as well as GenomicFeatures (R package, version 1.40.1, https://bioconductor.org/packages/release/bioc/html/GenomicFeatures.html) [32]. All of these sequence features were systematically investigated and compared between the SA-lncRNAs and SNA-lncRNAs. We utilized expression profile from five different tissues (leaf, flower, root, seed, and stem) to investigate the tissue-specificity of both SA-lncRNAs and SNA-lncRNAs. The fractional expression of each lncRNA in a particular tissue was calculated as its proportion relative to the total expression of that lncRNA across all the tissues [22]. The maximum fractional expression for each lncRNA was defined as its tissue-specific score across all the tissues. LncRNAs with a tissue-specific score greater than 0.6 in the leaf tissue were identified as leaf-specific lncRNAs [22].

### Expression correlation analysis of the lncRNAs between short-term and long-term salt treatment

To investigate the correlation between gene expressions of the lncRNAs identified from the short- and long-term salt treated leaves, the Pearson correlation coefficient of the lncRNAs between the short-term (24hvsCK and 48hvsCK) and long-term (14dvsCK and 28dvsCK) groups was calculated depending on their expression pattern changes between different salt stress-treated time spans. Pearson correlation coefficient was conducted by the cor.test() function of the R stats package.

### Target prediction and functional annotation of salt-associated lncRNAs in leaf

The expression levels of lncRNAs usually presented tight correlation with their potential target genes, suggesting that the lncRNAs may be likely to exhibit co-expression and related biological functions with their target mRNAs. Therefore, the underlying functions of SA-lncRNAs in *Avicennia marina* were analysed via the functional annotation and enrichment of their co-expressed mRNAs. In order to identify the co-expressed mRNAs for every lncRNA, Pearson correlations between the salt-associated lncRNAs and mRNAs were calculated [31]. As a result, the mRNAs that displayed relatively high correlations (> 0.5) and reliability (adjusted *p*-value < 0.05) with related lncRNAs were recognized as their co-expression mRNAs. Subsequently, the lncRNAs were respectively annotated by performing GO (Gene Ontology, biological process category) and KEGG (Kyoto Encyclopedia of Genes and Genomes) annotation for their co-expression mRNAs with the interproscan software (version 5.48–83.0) [33]. Functional annotations of each lncRNA were following carried out by conducting enrichment analysis for their correlated mRNAs with Hypergeometric test [34]. Top 20 enriched biological processes (BP) and KEGG pathways were apart selected for visualization when conducting the functional analysis of upregulated and downregulated SA-lncRNAs. The functional network was displayed by the Cytoscape (version 3.8.2) [35].

## Results

### Identification of salt-associated lncRNAs in *Avicennia marima* leaves

**Fig. 1:**
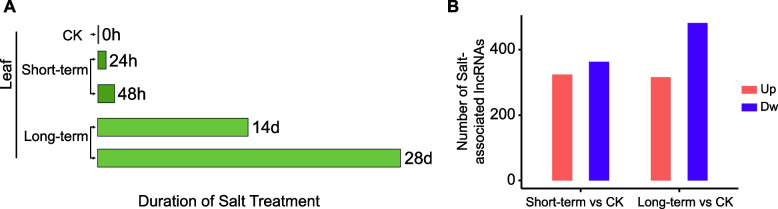
The identification of salt-associated long non-coding RNAs in the leaf of *Avicennia marina*. **A **The information for the short-term and long-term salt treatment. **B **The upregulated and downregulated short-term and long-term salt-associated lncRNAs. Up: upregulated; Dw: Downregulated; CK: Control

To investigate the long non-coding RNAs and their biological functions concerned to salt stress in *Avicennia marina*, we utilized RNA-seq data from the leaves respectively subjected to short- and long-term salt treatments to identify SA-lncRNAs (Fig. 1A). We compared the short-term (24 h and 48 h) treatment data with the control (CK) and identified 687 short-term SA-lncRNAs in the leaves (Fig. 1B and Table S1). Among them, 324 short-term SA-lncRNAs were upregulated under salt treatment, while 363 were inhibited by salinity treatment. By contrasting the lncRNAs of the long-term (14d and 28d) treatment samples with CK, we identified 797 long-term SA-lncRNAs, including 316 upregulated, and 481 downregulated members (Fig. 1B and Table S2). Notably, more downregulated SA-lncRNAs were identified than upregulated ones from both short- and long-term salt treated samples. This suggests that more downregulated SA-lncRNAs tend to be involved in the salt treatment responses, indicating their significant roles under salinity environment.

### Chromosome distribution of salt-associated leaf lncRNAs

**Fig. 2:**
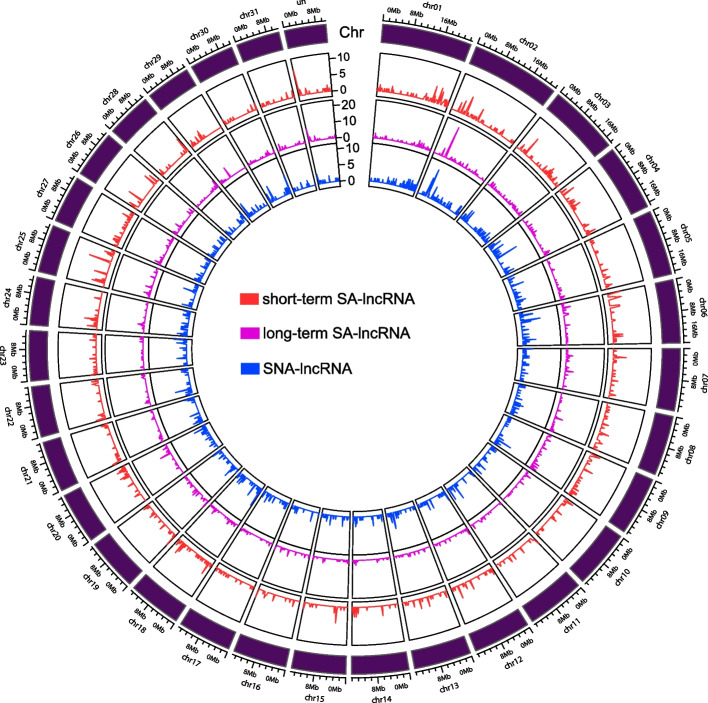
The chromosome distribution for the short-term and long-term salt-associated lncRNAs. The out layer indicates the chromosomes. The red, pink, and blue colors indicate the short-term salt-associated lncRNAs (short-term SA-lncRNAs), long-term salt-associated lncRNAs (long-term SA-lncRNAs), and salt-non-associated lncRNAs (SNA-lncRNAs), respectively

To examine the chromosome distribution of both short-term and long-term SA-lncRNAs identified in leaf tissue, we calculated the number of them on each chromosome. We observed that a significant number of leaf-salt-associated lncRNAs are distributed on chromosomes chr01 and chr02 (Figure S1A, B). Similarly, many salt-non-associated lncRNAs (SNA-lncRNAs) are also found on chr01 and chr02 (Figure S1C). This suggests that SA-lncRNAs and SNA-lncRNAs tend to exhibit similar chromosomal distributions. Furthermore, through the sliding-window analysis conducted among the chromosomes, we found that the distributions of short-term SA-lncRNAs and long-term SA-lncRNAs are consistent with those of SNA-lncRNAs on chromosomes such as chr01, chr02, chr03, chr04, etc. (Fig. 2). This further emphasizes the strong relevance between SA-lncRNAs and SNA-lncRNAs in terms of their chromosome assignments.

### Characteristics of salt-associated lncRNAs

**Fig. 3:**
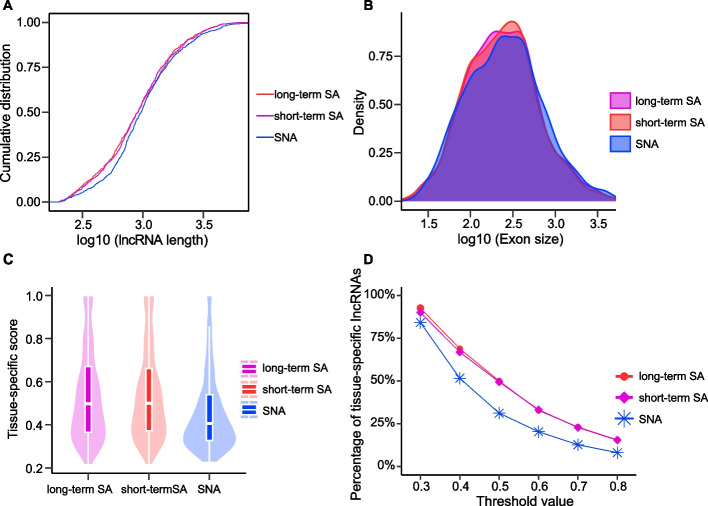
Characteristics of salt-associated lncRNAs for leaf in *Avicennia marina*. **A **The cumulative distribution of lncRNA length. **B **Density distribution of exon size. **C **Tissue-specific score in the short-term salt-associated lncRNAs (short-term SA-lncRNAs), long-term salt-associated lncRNAs (long-term SA-lncRNAs), and salt-non-associated lncRNAs (SNA-lncRNAs). **D **Percentage of tissue-specific salt-associated lncRNAs under varying thresholds of the tissue-specific score

To characterize the SA-lncRNAs in leaf tissue, we examined several features, including sequence length, exon number, exon size, tissue specificity, etc. of SA-lncRNAs and compared them with those of SNA-lncRNAs. We discovered that both the short-term SA-lncRNAs and long-term SA-lncRNAs possess slightly longer sequence length, larger exon, but smaller intron than SNA-lncRNAs (Fig. 3A, B and Figure S2A). However, there was no obvious difference observed in exon number between SA-lncRNAs and SNA-lncRNAs (Figure S2B).

As many lncRNAs function as regulators in a tissue-specific manner. To determine whether SA-lncRNAs play a tissue-specific role in leaf tissue development or tissue-related salt stress response, we calculated tissue-specific scores for SA-lncRNAs and compared them with those of SNA-lncRNAs. Then, we found that both the short-term SA-lncRNAs and long-term SA-lncRNAs show higher tissue-specific scores than SNA-lncRNAs (Fig. 3C). Regardless of the cut-off used for defining tissue-specific lncRNAs, SA-lncRNAs consistently displayed a higher percentage of tissue-specific lncRNAs compared to SNA-lncRNAs (Fig. 3D). Notably, SA-lncRNAs are predominantly expressed in leaf tissue, further underscoring their crucially salt-responsive regulatory role in leaves (Figure S2C-E).

### Target prediction and functional annotation of short-term salt-associated lncRNAs

**Fig. 4:**
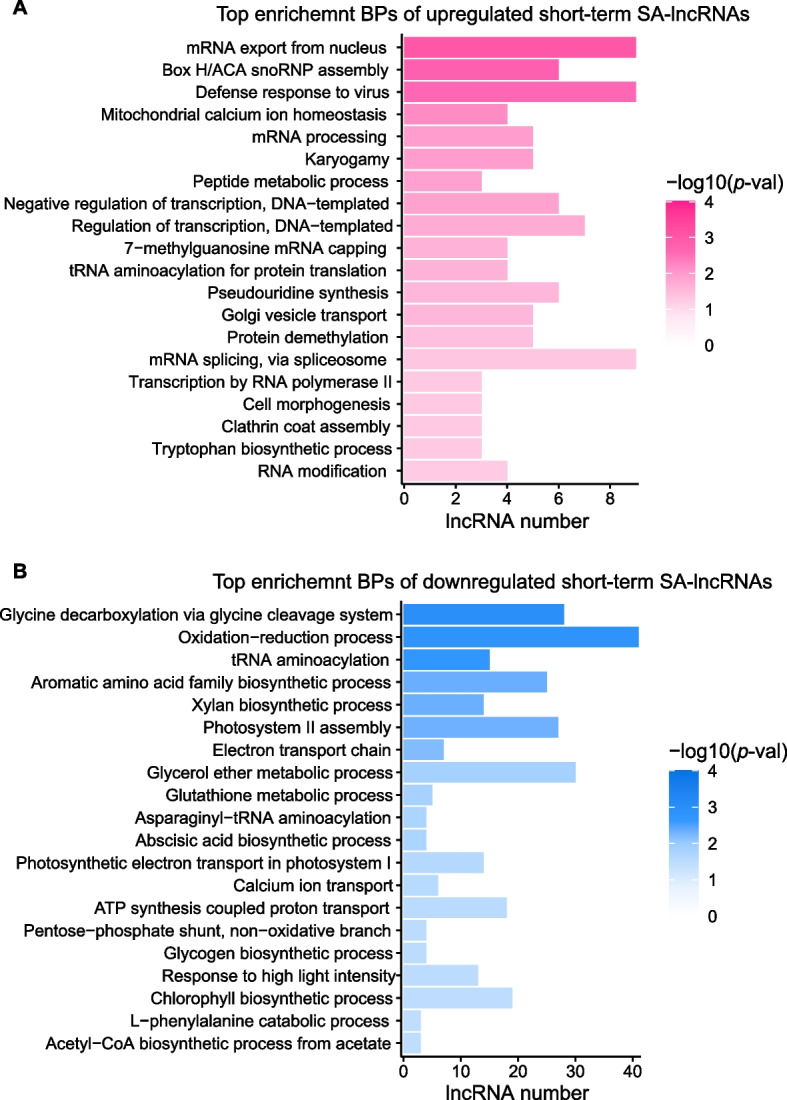
Functional enrichment analysis for the short-term salt-associated lncRNAs. **A**, **B **Top enrichment biological processes for the (**A**) upregulated and (**B**) downregulated short-term salt-associated lncRNAs (short-term SA-lncRNAs)

To examine the functional roles of short-term SA-lncRNAs, we conducted co-expression analysis between lncRNAs and mRNAs, and then used the co-expression mRNAs of lncRNAs to predict their potential functions. We discovered that the upregulated short-term SA-lncRNAs are enriched in the biological processes such as mRNA export from the nucleus, box H/ACA snoRNP assembly, defense response to virus, and mitochondrial calcium ion homeostasis and others (Fig. 4A). Contrarily, the downregulated short-term SA-lncRNAs are enriched in processes such as glycine decarboxylation via glycine cleavage system, oxidation–reduction process, calcium ion transport, photosystem II assembly, and others (Fig. 4B). Notably, the oxidation–reduction process and ion transport usually present strong association with abiotic stresses such as salt stress, suggesting that downregulated short-term SA-lncRNAs may play a role in regulating salt stress responses in the leaves of *Avicennia marina*.

### Target prediction and functional annotation of long-term salt-associated lncRNAs

The functional analysis of short-term SA-lncRNAs reveals that downregulated short-term SA-lncRNAs may play important roles in the oxidation − reduction process (Fig. 4B). Furthermore, we sought to determine which biological processes are likely to be regulated by long-term SA-lncRNAs. We applied the same method used for short-term associated lncRNAs to conduct functional analysis for the long-term SA-lncRNAs.

**Fig. 5:**
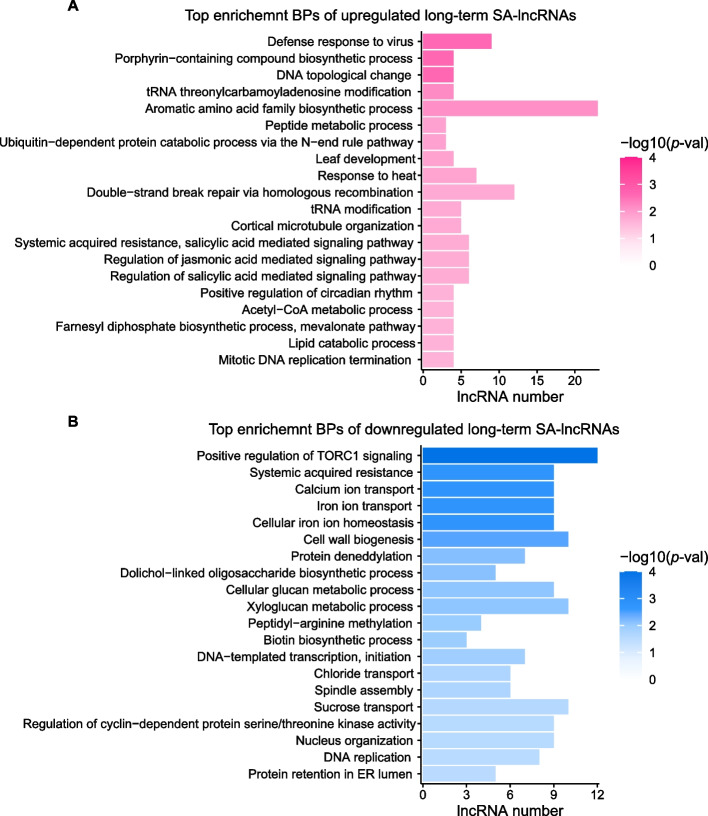
Functional enrichment analysis for the long-term salt-associated lncRNAs. **A**, **B **Top enrichment biological processes for the (**A**) upregulated and (**B**) downregulated long-term salt-associated lncRNAs (long-term SA-lncRNAs)

Interestingly, the biological processes enriched for the long-term SA-lncRNAs were similar to those of short-term SA-lncRNAs. The upregulated long-term SA-lncRNAs were significantly enriched in the following biological processes such as defense response to virus, leaf development, response to heat, regulation of salicylic acid and jasmonic acid mediated signaling pathways (Fig. 5A). This suggests that the upregulated long-term SA-lncRNAs potentially play their biological roles in modulating both abio-/ biotic stresses responses such as virus and temperature responses, leaf development, as well as phytohormone related stress responses. In addition, the downregulated long-term salt-associated lncRNAs were enriched in processes like positive regulation of TORC1 signaling, systemic acquired resistance, calcium ion transport, and iron ion transport as well as homeostasis, and others (Fig. 5B).

### Expression correlations between short-term and long-term salt treatment

**Fig. 6:**
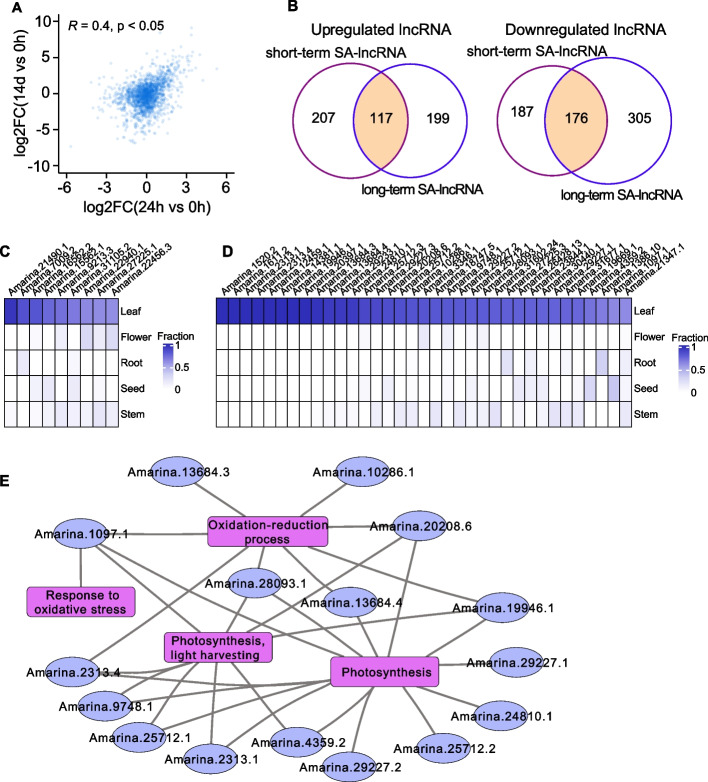
Cross talk analysis for the short-term and long-term salt associated lncRNAs. **A **Expression change (log2FoldChange) correlation between 14 days (14d) vs. CK and 24 h vs. CK. The correlation coefficient was calculated by the Pearson's correlation. **B **The overlap lncRNAs between the short-term and long-term salt-associated lncRNAs. **C **The concurrently upregulated salt-associated lncRNAs with leaf-specific expression. **D **The synchronously downregulated salt-associated lncRNAs with leaf-specific expression. **E **The synchronously downregulated salt-associated lncRNAs with leaf-specific expression are involved in the oxidation–reduction process and photosynthesis. In the visualization, lncRNAs are represented in blue, while the biological processes are depicted in pink

As the vanguard of the coastline, the habitat of *Avicennia marine* changed according to the tide. This implied that both the short- and long-term salt stresses are the abiotic stresses that should not be overlooked for *Avicennia marina*. To examine whether these two kinds of stresses have similar influences on the expression patterns of lncRNAs, we calculated the correlation of expression pattern changes of lncRNAs using the transcriptomic data from both short-term and long-term salinity treated leaf tissues. And we observed a strong correlation in expression variations between short-term and long-term salt treatment (Fig. 6A and Figure S3). Moreover, the KEGG enrichment results of the short-term SA-lncRNAs and long-term SA-lncRNAs indicated that the SA-lncRNAs were involved in the flavonoids biosynthesis, amino acid metabolism, lipids metabolism, terpenoids synthesis, staurosporine biosynthesis, etc. pathways (Figure S4). These results suggesting that plants may share the similar mechanisms in responding to salinity stress, whether it was short-term or long-term salt stress.

Furthermore, as it was shown in Fig. 6B, most SA-lncRNAs were identified both in the short-term and long-term salt-treated samples, as 117 upregulated lncRNAs were overlapped in the short- and long-term treatment, while 176 downregulated lncRNAs were found overlapped in the two treatments. Interestingly, we found that the concurrently downregulated SA-lncRNAs contained more leaf-specific lncRNAs than the upregulated SA-lncRNAs (Fig. 6C, D). These results indicates that downregulated SA-lncRNAs both under long-term and short-term treatments may play a dominant role in salt response in the leaf.

**Fig. 7:**
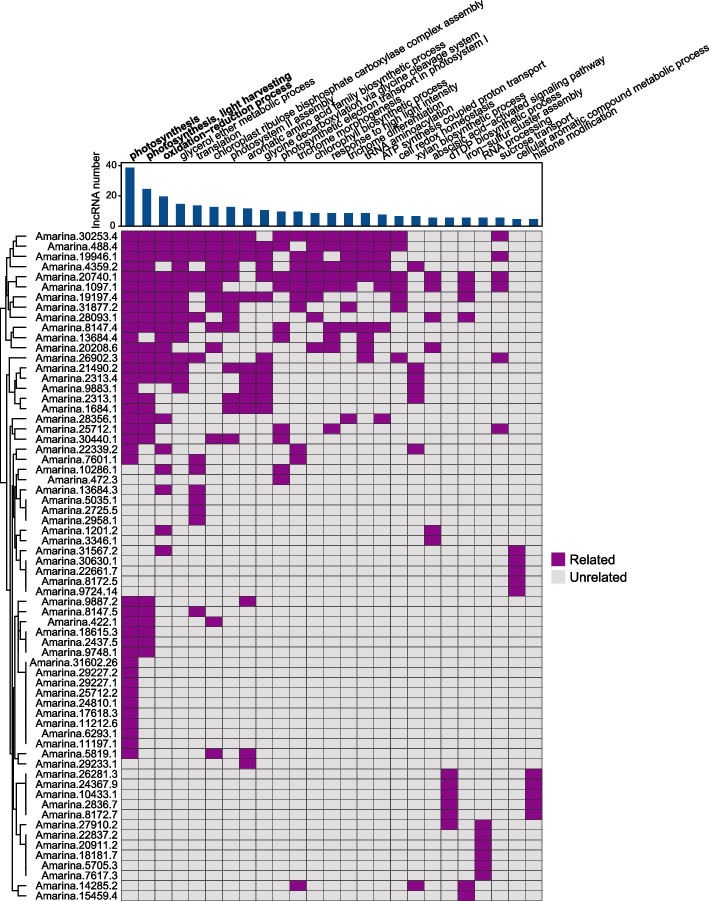
Top biological processes in relation to the synchronously downregulated lncRNAs. The biological processes are ranked by the number of its associated lncRNAs. Barplot indicates the number of synchronously downregulated lncRNAs

Leaf-specific SA-lncRNAs synchronously downregulated under long-term and short-term treatments are involved in processes such as the oxidation–reduction process (e.g., *Amarina.13684.3*, *Amarina.10286.1*, and *Amarina.20208.6*), response to oxidative stress (e.g., *Amarina.1097.1*), and photosynthesis (e.g., *Amarina.13684.4* and *Amarina.19946.1*), and others (Fig. 6E). Additionally, we found that the majority of 176 synchronously downregulated lncRNAs are also related to the biological processes such as photosynthesis, and oxidation–reduction process (Fig. 7). These results are consistent with those observations for the leaf-specific SA-lncRNAs that synchronously downregulated (Fig. 6E), which further suggests that these downregulated SA-lncRNAs potentially play a crucial role in controlling the salt stress response by regulating oxidation–reduction process and photosynthesis during short-term and long-term salt exposure.

## Discussion

*Avicennia marina* is a kind of mangrove occurred extensively worldwide, which can thrive in high salinity environment by absorbing and secreting Na^+^ [36]. The leaf is a crucial tissue for salt secretion and photosynthesis in *Avicennia marina* under salt stress. Long non-coding RNAs (lncRNAs) play a significant role in various biological processes, including salt response [37]. However, there have been no reports on the functional role of lncRNAs in the leaf under salt treatment for *Avicennia marina.*

In this present study, utilizing RNA-sequencing data from short-term and long-term salt treatments, we identified 687 short-term and 797 long-term salt-associated lncRNAs. Similarly, in *Populus trichocarpa*, 1054 and 765 differentially expressed lncRNAs were severally identified from three tissues under 7d and 24 h salt stress treatments [27] suggesting that lncRNAs were likely to play vital parts during salinity stress response. While, the number of salt-responsive lncRNAs found in *Avicennia marina* is less than that in *Populus trichocarpa*, *Medicago truncatula* and other plants, this is likely to be resulted from the differences of plant species, including their sensitivities to salt stress and genome sizes [38–41]. These SA-lncRNAs exhibit slight differences in characteristics, including length, exon size, intron size, and tissue specificity, when compared to SNA-lncRNAs. This suggests that SA-lncRNAs possess unique features in per sequence in comparison to the SNA-lncRNAs, which may imply the differentially biological functions of salt-associated and salt-non-associated lncRNAs.

Additionally, correlation analysis results of the expression changes of the SA-lncRNAs reveals that short-term and long-term salt treatments may have similar effects on lncRNAs’ expressions. Furthermore, the majority of SA-lncRNAs are identified in both short-term and long-term salt treatments, indicating that the responsive mechanisms to salt stress may be similar when *Avicennia marina* under either long-term or short-term salt stress, and the expression of lncRNAs are potentially regulated as well as respond to salt stress in a tissue-oriented mode [27, 40]. Many of the commonly downregulated SA-lncRNAs are leaf-specific and involved in processes related to oxidation–reduction and photosynthesis, as well as the metabolic pathways as flavonoids biosynthesis, amino acid metabolism, lipids metabolism, terpenoids synthesis pathways. The functional annotation of SA-lncRNAs in this study keeps consistent with the previous research that the lncRNA neighbouring co-expression genes were significantly enriched in the defence response, oxidation–reduction process and metabolic processes, including flavonoids, amino acid, lipids and terpenoids [27, 38, 42, 43]. This implies that SA-lncRNAs may regulate short-term and long-term salt responses through the regulation of the oxidation–reduction process and photosynthesis as well as the involving in several metabolic processes [41, 44].

## Conclusion

As a coastal halophyte, the *Avicennia marina* can adapt to high salinity environment. However, previously research is mainly focused on the physiological and biochemical research and the molecular mechanism is largely unknown. Long non-coding RNAs (lncRNAs) play a significant role in various biological processes, including salt response, while their functions are still in its infancy.

In this study, we identified the salt-associated lncRNAs from the leaf samples under salinity treatment, and conducted feature and functional analyses of them. By conducting a comprehensive functional analysis of SA-lncRNAs in *Avicennia marina* under various salinity treatments, we got a preliminary understanding of the functions of SA-lncRNAs in leaf tissue. Generally speaking, this research aims to provide a better understanding of lncRNAs’ function in the salinity stress of leaf tissue, ultimately contribute to our background knowledge about its regulatory role. And finally, provide us new insights to understand the salt-responsive mechanisms of *Avicennia marina* as well as other plant species.

### Supplementary Information

Additional file 1: Table S1. Short-term salt-associated lncRNAs in the leaf.

Additional file 2: Table S2. Long-term salt-associated lncRNAs in the leaf.

Additional file 3: Fig. S1: The number of salt-associated lncRNAs across the chromosome. (A) The number of short-term salt-associated lncRNAs across the chromosome. (B) The number of long-term salt-associated lncRNAs across the chromosome. (C) Number of salt-non-associated lncRNAs in each chromosome.

Additional file 4: Fig. S2: Intro size, exon number, tissue specificity between the salt-associated and salt-non-associated lncRNAs in the leaf. (A) The cumulative distribution of intro size. (B) Exon number in the short-term salt-associated, long-term salt-associated, salt-non-associated lncRNAs. (C-E) Heatmap of the fractional value across the tested tissues for the (C) short-term salt-associated, (D) long-term salt-associated, (E) salt-non-associated lncRNAs.

Additional file 5: Fig. S3: Expression change (log2FoldChange) correlation between short-term and long-term salt treatment. (A) Expression change correlation between 28days (28d) vs. CK and 24hours (24h) vs. CK. (B) Expression change correlation between 14days (14d) vs. CK and 48hours (48h) vs. CK. (C) Expression change correlation between 28days (28d) vs. CK and 48hours (48h) vs. CK.

Additional file 6: Fig. S4: KEGG enrichment for the SA-lncRNAs under short-term and long-term salt treatment. (A) Top KEGG enrichment pathways of short-term salt-associated lncRNAs. (B) Top KEGG enrichment pathways of long-term salt-associated lncRNAs.

## Data Availability

The data is available in Supplementary material. Meanwhile, the lncRNAs (gtf file) analyzed during the current study are available in the github repository (https://github.com/wangll0207/Avicennia_marina.LncRNA).

## Abbreviations

lncRNA: Long non-coding RNA
FPKM: Fragments Per Kilobase of sequence per Million mapped reads
short-term SA-lncRNAs: Short-term salt-associated lncRNAs
long-term SA-lncRNAs: Long-term salt-associated lncRNAs
SNA-lncRNA: Salt-non-associated lncRNAs
