# Supplementary figures and images for: Identification and analysis of short-term and long-term salt-associated lncRNAs in the leaf of Avicennia marina
Source: BMC Plant Biol. 2024 Jun 5;24:500. doi: 10.1186/s12870-024-05216-z (PMC11151563; doi:10.1186/s12870-024-05216-z)

Figure S1

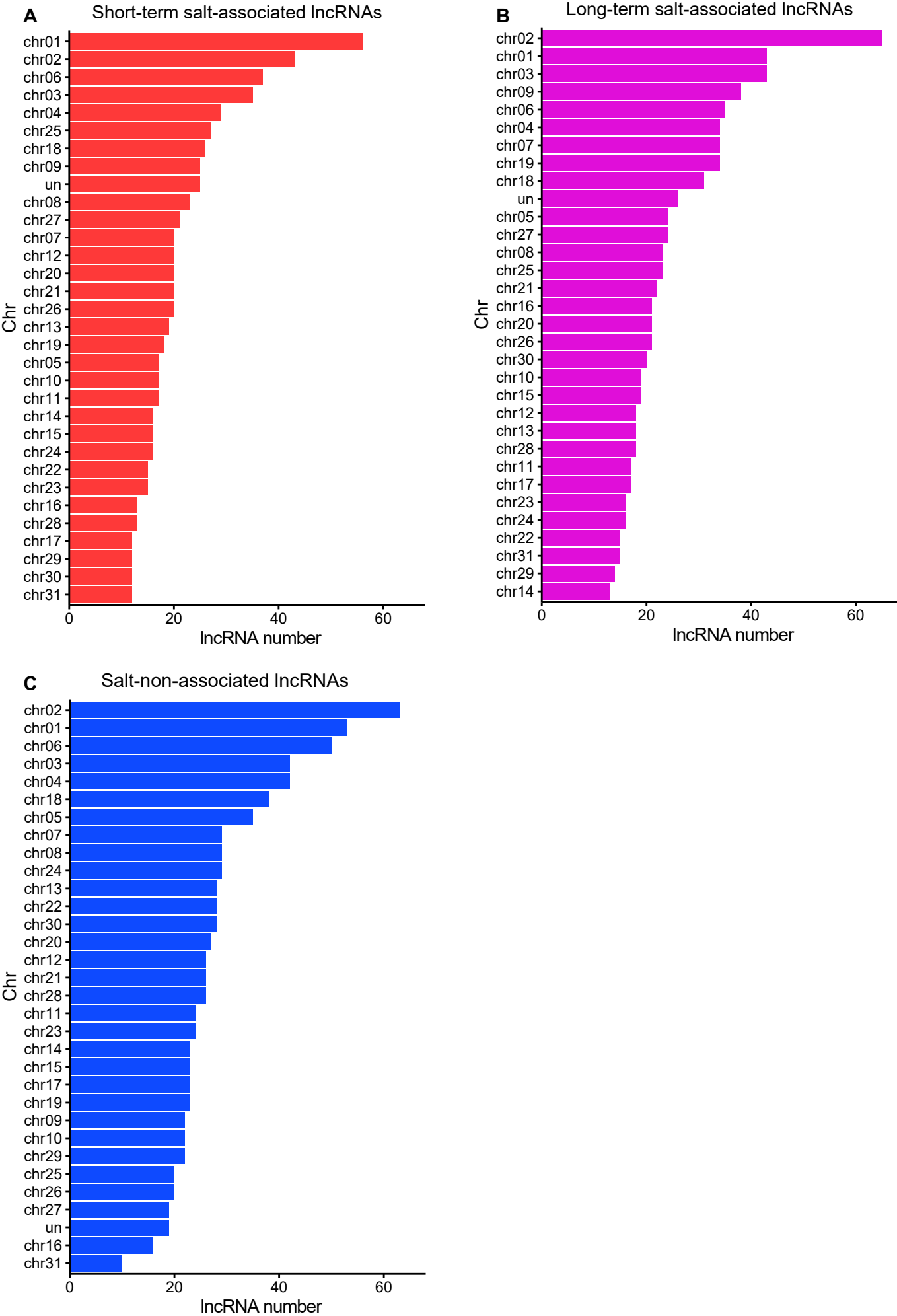

Supplement: Supplementary file 3 — Additional file 3: Fig. S1: The number of salt-associated lncRNAs across the chromosome. (A) The number of short-term salt-associated lncRNAs across the chromosome. (B) The number of long-term salt-associated lncRNAs across the chromosome. (C) Number of salt-non-associated lncRNAs in each chromosome. [file 12870_2024_5216_MOESM3_ESM.pdf]

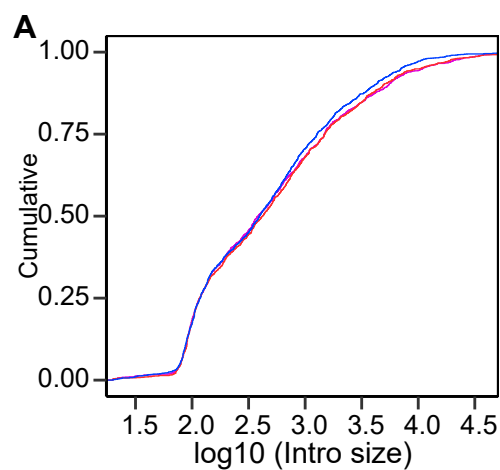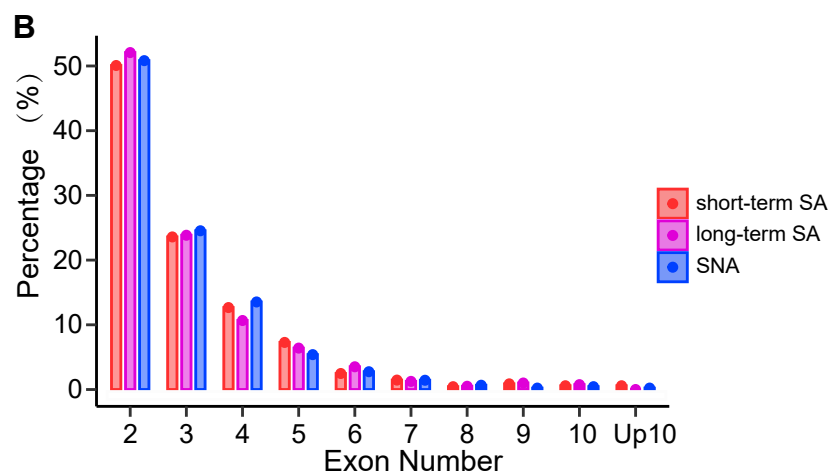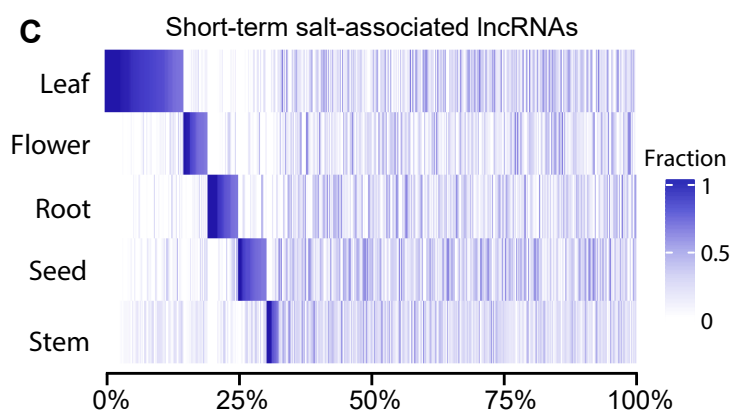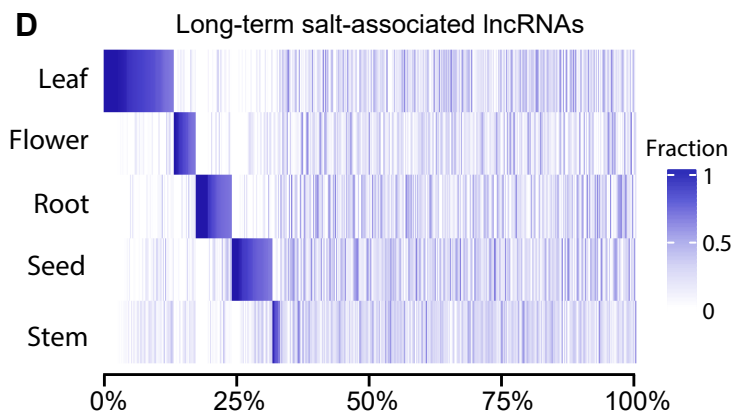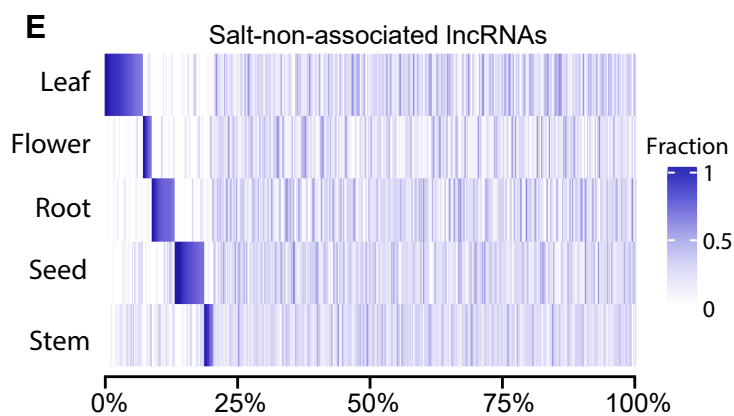

Supplement: Supplementary file 4 — Additional file 4: Fig. S2: Intro size, exon number, tissue specificity between the salt-associated and salt-non-associated lncRNAs in the leaf. (A) The cumulative distribution of intro size. (B) Exon number in the short-term salt-associated, long-term salt-associated, salt-non-associated lncRNAs. (C-E) Heatmap of the fractional value across the tested tissues for the (C) short-term salt-associated, (D) long-term salt-associated, (E) salt-non-associated lncRNAs. [file 12870_2024_5216_MOESM4_ESM.pdf]

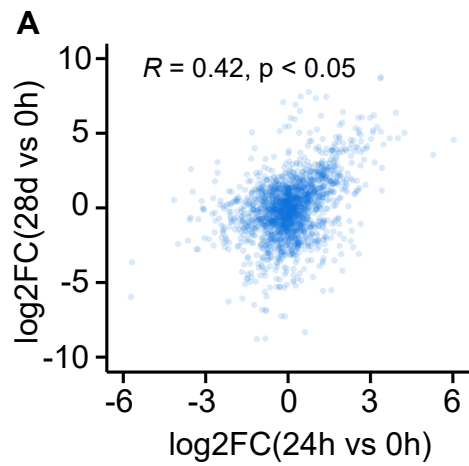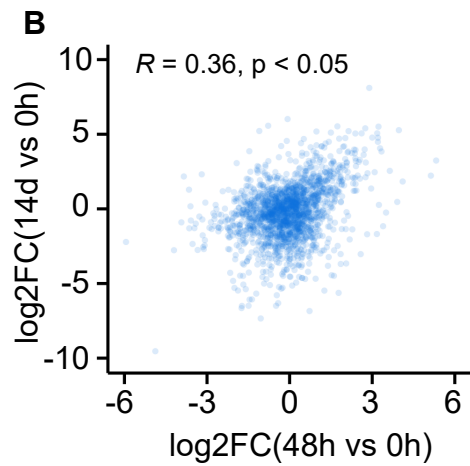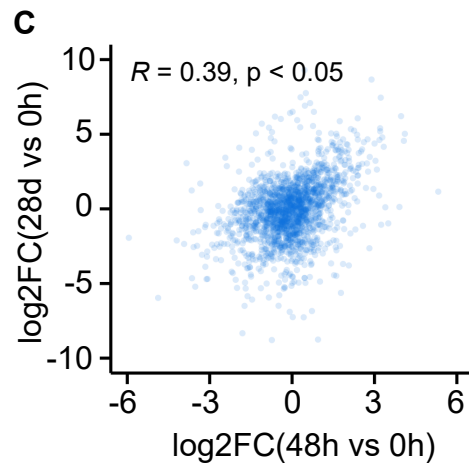

Supplement: Supplementary file 5 — Additional file 5: Fig. S3: Expression change (log2FoldChange) correlation between short-term and long-term salt treatment. (A) Expression change correlation between 28days (28d) vs. CK and 24hours (24h) vs. CK. (B) Expression change correlation between 14days (14d) vs. CK and 48hours (48h) vs. CK. (C) Expression change correlation between 28days (28d) vs. CK and 48hours (48h) vs. CK. [file 12870_2024_5216_MOESM5_ESM.pdf]
