# Supplementary material for: Identification and analysis of short-term and long-term salt-associated lncRNAs in the leaf of Avicennia marina
Source: BMC Plant Biol. 2024 Jun 5;24:500. doi: 10.1186/s12870-024-05216-z (PMC11151563; doi:10.1186/s12870-024-05216-z)
Supplement: Supplementary file 6 — Additional file 6: Fig. S4: KEGG enrichment for the SA-lncRNAs under short-term and long-term salt treatment. (A) Top KEGG enrichment pathways of short-term salt-associated lncRNAs. (B) Top KEGG enrichment pathways of long-term salt-associated lncRNAs. [file 12870_2024_5216_MOESM6_ESM.pdf]

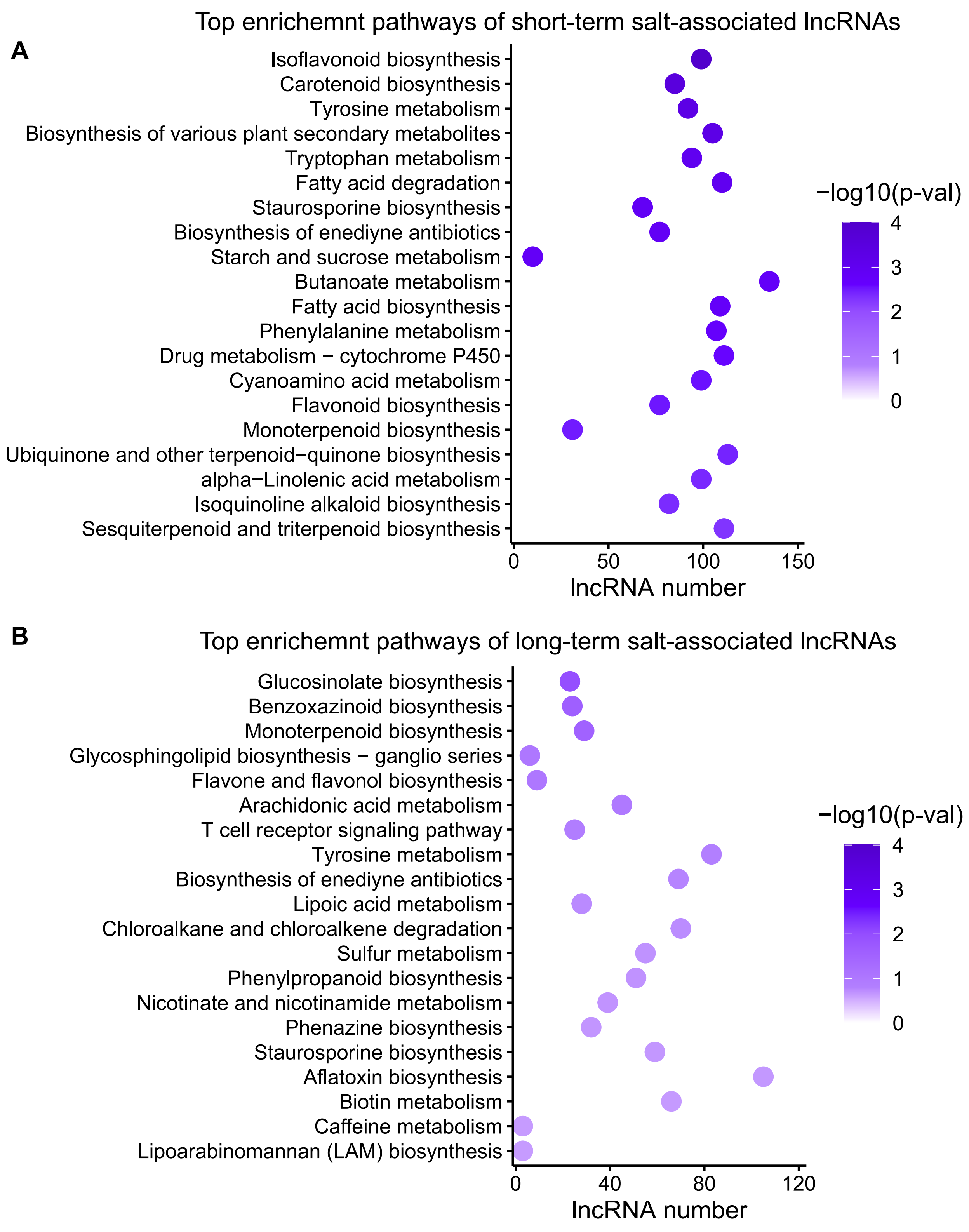
A
Top enrichemnt pathways of short-term salt-associated lncRNAs
Isoflavonoid biosynthesis
Carotenoid biosynthesis
Tyrosine metabolism
Biosynthesis of various plant secondary metabolites
Tryptophan metabolism
Fatty acid degradation
Staurosporine biosynthesis
Biosynthesis of enediyne antibiotics
Starch and sucrose metabolism
Butanoate metabolism
Fatty acid biosynthesis
Phenylalanine metabolism
Drug metabolism − cytochrome P450
Cyanoamino acid metabolism
Flavonoid biosynthesis
Monoterpenoid biosynthesis
Ubiquinone and other terpenoid−quinone biosynthesis
alpha−Linolenic acid metabolism
Isoquinoline alkaloid biosynthesis
Sesquiterpenoid and triterpenoid biosynthesis
0
50
100
150
lncRNA number
−log10(p-val)
4
3
2
1
0
B
Top enrichemnt pathways of long-term salt-associated lncRNAs
Glucosinolate biosynthesis
Benzoxazinoid biosynthesis
Monoterpenoid biosynthesis
Glycosphingolipid biosynthesis − ganglio series
Flavone and flavonol biosynthesis
Arachidonic acid metabolism
T cell receptor signaling pathway
Tyrosine metabolism
Biosynthesis of enediyne antibiotics
Lipoic acid metabolism
Chloroalkane and chloroalkene degradation
Sulfur metabolism
Phenylpropanoid biosynthesis
Nicotinate and nicotinamide metabolism
Phenazine biosynthesis
Staurosporine biosynthesis
Aflatoxin biosynthesis
Biotin metabolism
Caffeine metabolism
Lipoarabinomannan (LAM) biosynthesis
0
40
80
120
lncRNA number
−log10(p-val)
4
3
2
1
0
